# Proteomic landscape subtype and clinical prognosis of patients with the cognitive impairment by Japanese encephalitis infection

**DOI:** 10.1186/s12974-022-02439-5

**Published:** 2022-04-04

**Authors:** Rong Yin, Linpeng Yang, Ying Hao, Zhiqi Yang, Tao Lu, Wanjun Jin, Meiling Dan, Liang Peng, Yingjie Zhang, Yaxuan Wei, Rong Li, Huiping Ma, Yuanyuan Shi, Pengcheng Fan

**Affiliations:** 1 Department of Neurology, Lanzhou General Hospital, Lanzhou, 730050 China; 2 Department of Neurology, Gansu Province Central Hospital, Lanzhou, 730070 China; 3 Department of Pharmacy, Lanzhou General Hospital, Lanzhou, 730050 China; 4 The Fourth Department of Research, Center for Gansu Provincial Vaccine Engineering Research, Lanzhou, 730046 China; 5 Pathology and Laboratory Medicine, Weill Cornell Medical College, New York, 10065 USA; 6 Department of Neurology, Lanzhou University Second Hospital, Lanzhou, 730030 China; 7 School of Life Sciences, Beijing University of Chinese Medicine, Beijing, 102488 China; 8 Department of Neurology, Chongqing University Fuling Hospital, Chongqing, 408000 China; 9 School of Life Sciences, Lanzhou University, Lanzhou, 730000 China; 10 The First Clinical Medical School, Gansu University of Chinese Medicine, Lanzhou, 730030 China; 11 Shenzhen Research Institute, Beijing University of Chinese Medicine, Shenzhen, 518118 China; 12 State Key Laboratory of Proteomics, National Center for Protein Sciences (Beijing), Institute of Lifeomics, Beijing, 102206 China

**Keywords:** Japanese encephalitis, Cerebrospinal fluid, Proteomics, Dementia, Cognitive impairment

## Abstract

**Background:**

Cognitive impairment is one of the primary sequelae affecting the quality of life of patients with Japanese encephalitis (JE). The clinical treatment is mainly focused on life support, lacking of targeted treatment strategy.

**Methods:**

A cerebrospinal fluid (CSF) proteomic profiling study was performed including 26 patients with JE in Gansu province of China from June 2017 to October 2018 and 33 other concurrent hospitalized patients who were excluded central nervous system (CNS) organic or CNS infection diseases. The clinical and proteomics data of patients with JE were undergoing combined analysis for the first time.

**Results:**

Two subtypes of JE associated with significantly different prognoses were identified. Compared to JE1, the JE2 subtype is associated with lower overall survival rate and a higher risk of cognitive impairment. The percentages of neutrophils (N%), lymphocyte (L%), and monocytes (M%) decreased in JE2 significantly.

**Conclusions:**

The differences in proteomic landscape between JE subgroups have specificity for the prognosis of cognitive impairment. The data also provided some potential target proteins for treatment of cognitive impairments caused by JE.

*Trial registration* ChiCTR, ChiCTR2000030499. Registered 1st June 2017, http://www.medresman.org.cn/pub/cn/proj/projectshow.aspx?proj=6333

**Supplementary Information:**

The online version contains supplementary material available at 10.1186/s12974-022-02439-5.

## Background

Japanese encephalitis (JE) is an acute infectious disease caused by the JE virus (JEV), which is often transmitted by mosquito bites [1]. The prognosis is often poor when clinical manifestations occur, with one-third of infected individuals retaining neurological sequelae [2]. Cognitive impairment is one of the primary factors affecting the quality of life of patients with JE [3, 4]. Few studies have explored the relationship between cerebrospinal fluid (CSF) protein changes and prognosis in patients with JE [5, 6]. The causes of neurological sequelae of JE—especially cognitive impairment—remain unclear. The JEV primarily targets neurons and microglia, causing a high degree of inflammation in the central nervous system (CNS) and leading to substantial neuronal damage or dysfunction [7]. The functional and structural integrity of the blood–brain barrier (BBB) are also damaged [8]. Imaging examinations and autopsies show that JEV often injures the basal ganglia, putamen, thalamus, substantia nigra, brainstem, hippocampus, cerebral cortex, and spinal cord, causing a series of serious CNS symptoms including acute flaccid paralysis, aseptic meningitis, and encephalitis [5, 6]. Some severe patients develop illness onset rapidly, leading to respiratory failure, poor prognosis and high mortality. Some surviving patients also display neurological sequelae with unclear reason [4]. The current study is the first clinical research to describe the relationship of cognitive impairment with proteomics profiling changes of CSF in patients with JE. These patients are divided into two subtype by proteomics profiling. Patients with JE2 molecular subtyping showed poor prognosis along with severe cognitive impairment.

## Materials and methods

### Enrolment of patients with JE and collection of clinical data

Thirty-four patients diagnosed with JE were initially enrolled in this study. Eight patients were excluded, because not enough CSF samples were left after clinical examination to complete the proteomics study. Finally, this retrospective study included 26 patients with a confirmed diagnosis of JE, who were admitted in Department of Clinical Neurology, the Lanzhou General Hospital (Lanzhou, China) from June 1, 2017 to October 31, 2018. The 33 patients in the control group were enrolled from other concurrent hospitalized patients who were excluded central nervous system (CNS) organic or CNS infection diseases. The patients with JE included the patients in the acute stage of encephalitis. All JE cases were reported to the Gansu Sub-centre of the Centre of Disease Control in China. This study implemented the World Health Organization recommendations and defined JE based on the following criteria: presence of the clinical criteria of acute encephalitis syndrome and (1) detectable JE-specific IgM in the CSF or serum, or (2) evidence of seroconversion or a fourfold increase in IgM or IgG in the convalescence phase as detected by the ELISA method according to the diagnostic guidelines, or (3) isolation of the virus from the blood, CSF fluid, or tissue, or (4) detection of the JE virus genome in the serum, plasma, blood, CSF, or tissue. The inclusion and exclusion criteria are described in the Chinese Clinical Trial Registry (ChiCTR2000030499, http://www.chictr.org.cn/). General and special clinical information, CSF tests and intracranial pressure, head magnetic resonance imaging (MRI), Glasgow Coma Scale (GCS) score, Mini-Mental State Exam (MMSE) score (before discharge), and mRS (1 month after discharge) were collected. This study was approved by the Ethics Committee of the Lanzhou General Hospital prior to initiation (2017XYLL050). Consent forms were signed by all patients or their legal guardians.

### Clinical sample detection

Acute-phase serum was obtained within 7 days after the onset of symptoms and all CSF samples were collected within 48 h after admission to hospital. Serum and CSF were collected and immediately sent for examination. The blood routine examination and CSF samples detection were tested by the BC-6900 Laboratory Hematology System automatic blood cell analyser (Mindray, Shenzhen, China). The detection included white blood cell (WBC), red blood cell (RBC), the percentages of neutrophils (N%), lymphocyte (L%), and monocytes (M%).

### CSF sample collection and preparation

For patients who required clinical CSF examination, strictly limited volumes of CSF were obtained through lumbar puncture performed in the Department of Neurology following clinical guidelines. The CSF samples were immediately stored at 4 °C, and the clinical exam and laboratory tests were completed within 4 h. After clinical tests the residual CSF samples were centrifuged at 16,000 *g* for 10 min, the supernatant was collected. The protein concentration was determined using the Bicinchoninic Acid protein assay. After centrifugation, the CSF was sub-packed and stored at – 80 °C. For sample identification, all samples were numbered with codes (such as C01, JE01, and so on) instead of the patients’ name and hospital ID.

### Protein in-gel digestion and LC–MS/MS analysis of the CSF peptides

For each sample, CSF containing approximately 100 μg of protein was digested according to the protein in-gel digestion protocol [9]. After digestion for 12 h, the peptide digestion products were extracted. The supernatants were dried by rotary evaporation and stored at 4 °C. Approximately 1 μg of dried CSF peptide samples were analysed using a LC–MS/MS Orbitrap Fusion Lumos platform (Thermo Fisher Scientific, Rockford, IL, USA) comprising an Easy-nLC™ 1000 nanoflow LC system (Thermo Fisher Scientific). Data were acquired using the Xcalibur software (Thermo Fisher Scientific).

### Protein identification and protein quantification

The mass spectra raw files were searched against the UniProt human database (version 20180903; 20,386 sequences) using the MaxQuant software (version 1.6.2.3). Methionine oxidation and N-terminal acetylation were chosen as the variable modifications. Cysteine carbamidomethylation was chosen as the fixed modification, and trypsin was selected as the digestion enzyme. The mass spectra data was also searched against a decoy database. The false discovery rates of the peptide-spectrum matches and proteins were set to < 1%. Matches between runs were used to ensure the identifications were transferred to non-sequenced or non-identified MS features in all LC–MS runs. Proteins which had at least 2 unique peptides, were selected for further analysis. Label-free protein quantifications were calculated using a label-free, intensity-based absolute quantification (iBAQ) approach [10]. Proteome quantification was performed with the iBAQ algorithm followed by normalization to fraction of total (FOT) [11]. FOT was used to represent the normalised abundance of a particular protein across samples. It was defined as a protein’s iBAQ divided by the total iBAQ of all identified proteins within one sample. The FOT was multiplied by 10^5^ for ease of presentation. The cutoff criteria were set as proteins with at least two or more unique peptides, a quantification ratio (compared with mean of the control group) ≥ 3 or ≤ 0.33, *p* ≤ 0.05, and changed proteins identified in more than one third of all the samples, respectively.

### Proteome data statistical analysis

R version 3.5.1 was used for data analysis and data visualisation. PCA was used to visualise the separation of controls and JEs [12]. R packages ‘ggplot2’, ‘FactoMineR’, and ‘factoextra’ were used to visualise the PCA results. Different expressed proteins were identified by a volcano map. MeV software version 4.9.0 was used to perform heatmap analysis [13]. The protein–protein interactions and pathway alterations were identified by Reactome and String databases. JE proteomic subtype identification was performed by Hierarchical Clustering (HCL) analysis. The distance metric used for HCL was the Pearson correlation coefficient. Average linkage clustering was selected as the linkage method. Gene leaf order and sample leaf order were both optimised, and the correlation matrix between proteomic subtypes and clinical features were analysed by the ‘ggcorr’ function in the R package ‘GGally’ [14]. Functional enrichment analysis of signature proteins was performed using the Kyoto Encyclopedia of Genes and Genomes [15] and STRING [16].

### Western blot analysis

The samples in the control (*n* = 33), JE1 (*n* = 14), and JE2 (*n* = 12) groups were mixed into 12 independent samples randomly. The CSF total protein (TP) staining was used as loading control to improve the sensitivity and accuracy of CSF western blots results [17]. In this study, the grey level of TP SDS–PAGE band was used as the normalization of CSF loading control. For each sample, 20 μg of protein extracts from the previous step (See section CSF sample collection and preparation) was separated using 10% SDS–PAGE and the proteins were transferred onto polyvinylidene fluoride membranes. After blocking with 5% skim-milk solution in 1% tris buffered saline with Tween 20 (TBST, *w*/*v;* BD Biosciences, San Jose, CA, USA) for 2 h, the membranes were incubated with 5% skim-milk containing appropriate primary antibodies overnight at 4 °C. On the second day, the membranes were washed 4 times with 1 × TBST buffer followed by 2 h of incubation with horseradish peroxidase-conjugated secondary antibodies. Signals of target protein bands were detected using a chemiluminescent detection reagent. The Image J software was used to quantify the grey level of the band. The primary antibodies included antibodies SPARC-like 1 protein (SPARCL1) (ab107533, Abcam, Cambridge, UK), IgE (154/102) (sc-53346, Santa Cruz Biotechnology, Dallas, TX, USA), Igλ chain (A-3) (sc-166295, Santa Cruz Biotechnology, Dallas, TX, USA), Complement 5a (ab183597, Abcam, Cambridge, UK), ITIH4 (ab180139, Abcam, Cambridge, UK), Cystatin C (ab109508, Abcam, Cambridge, UK), and β-Amyloid (B-4) (sc-28365, Santa Cruz Biotechnology, Dallas, TX, USA) which were used in a 1:1000 dilution. The secondary antibodies included goat anti-rabbit IgG (ZDR 5118) and goat anti-mouse IgG (ZDR 5006).

## Results

### Demographic characteristics and clinical features of patients with JE

The demographics and clinical features of the 59 patients are shown in Additional file 7: Table S1. The most common symptoms in JE participants at initial presentation were fever (96.15%) and unconsciousness (73.08%). The median GCS score at initial encounter was 10 (range 3–15) and the central respiratory failure/tracheotomy rate was 46.15%. Moreover, mental health symptoms and seizures occurred in 18 (69.23%) and 3 (11.54%) patients, respectively. Sixteen patients developed lung infections, 13 developed limb paralysis, 12 presented pathological evidences, and 15 presented abnormal signals on cranial MRI. Four patients (mortality rate, 15.38%) died in the hospital. Before being discharged, most of the patients had severe cognitive impairment, and MMSE score was 15.7 ± 8.7. After 1 month, the Modified Rankin Score (mRS) was 1.58 ± 1.98, and 19 patients had a good outcome (mRS ≤ 2).

The blood routine examination revealed that the WBC count was slightly higher in the JE group (8.78 ± 2.72, × 10^9^·L^−1^) than in the control group (7.17 ± 3.17, × 10^9^·L^−1^). The RBC count (4.15 ± 0.56, × 10^12^·L^−1^) was not significantly changed. This trend is consistent with the results of a previous study that reported that the number of viral copies in circulation decline rapidly a few days after JEV infection [18]. The levels of procalcitonin (1.13 ± 2.23, μg·L^−1^) and blood glucose (7.22 ± 2.82, mmol·L^−1^) were slightly higher in the JE group.

CSF examination revealed that the WBC levels (111.23 ± 231.30, × 10^6^·L^−1^) were significantly higher in the JE group than in the control group (9.67 ± 8.13, × 10^6^·L^−1^). The CSF intracranial pressure, glucose, and chloride levels were all within normal range for patients with JE and those in the control group. The CSF protein concentration was higher in patients with JE (680.7 ± 284.4 mg·L^−1^) than in the control patients (329.9 ± 173.1 mg·L^−1^).

The most common symptom after JEV infection was fever (25 in 26, 96.15%). Consciousness disorder, mental symptoms, secondary pulmonary infection, limb paralysis, abnormal signals in MRI, pathological signs, and convulsions are the common clinical features of patients. Nearly half of patients with JE (12 in 26, 46.14%) progressed, developed rapid respiratory failure, and received mechanical ventilation with tracheotomy. The neurophilic characteristics of the JEV causes central respiratory failure in the early central infection stage [19–21]. The mortality rate of cases in our study was 15.4% (4 in 26), lower than the 20–30% reported by Solomon et al. [22], but higher than the 4.4% reported by Lo et al. [23]. Early mechanical ventilation and symptomatic treatment improved patients' recovery and reduced the mortality. Most of the hospitalised patients with JE were transferred from primary hospitals and were severe. The mortality rate was similar to those previously reported for severe cases [23]. The mRS at 30 days after discharge was 1.58 (0–2). In return investigations 1 month after discharge, most of the patients had recovered and were able to take care of themselves. Their nervous system function was also improved. Only four patients had a mRS score of 2. We found that the rate of cognitive decline was higher and the extent was severe, as previously reported [24, 25].

### The MS platform stability

The average correlation coefficient of the protein quantification results among repeated samples from different patients was 0.98 (range 0.97–1.00, Additional file 1: Fig. S1A), which is close to those reported previously [11, 26]. These results demonstrated the consistent stability of the MS platform.

### CSF proteome profiling

The mass spectrometry (MS) platform was stable and repeatable as judged by quality control runs during the entire data-collection period (Additional file 1: Fig. S1A). The effect of sample size on the identification of CSF peptides and proteins were investigated. When the number of CSF samples reached 40 cases, the number of identified peptides and proteins approached saturation (Additional file 1: Fig. S1B). In total, 2864 peptides and 398 proteins were identified. The effect of the amount of CSF sample on the identification of peptides and proteins were also investigated. When the protein (digestion peptides) loading amounted to approximately 0.5 μg, the protein identification number was close to saturation. Further increasing the sample loading amount had little effect on increasing the number of proteins identified (Additional file 1: Fig. S1C). The number of quantified proteins was similar in control and JE group (Additional file 1: Fig. S1D).

**Fig. 1:**
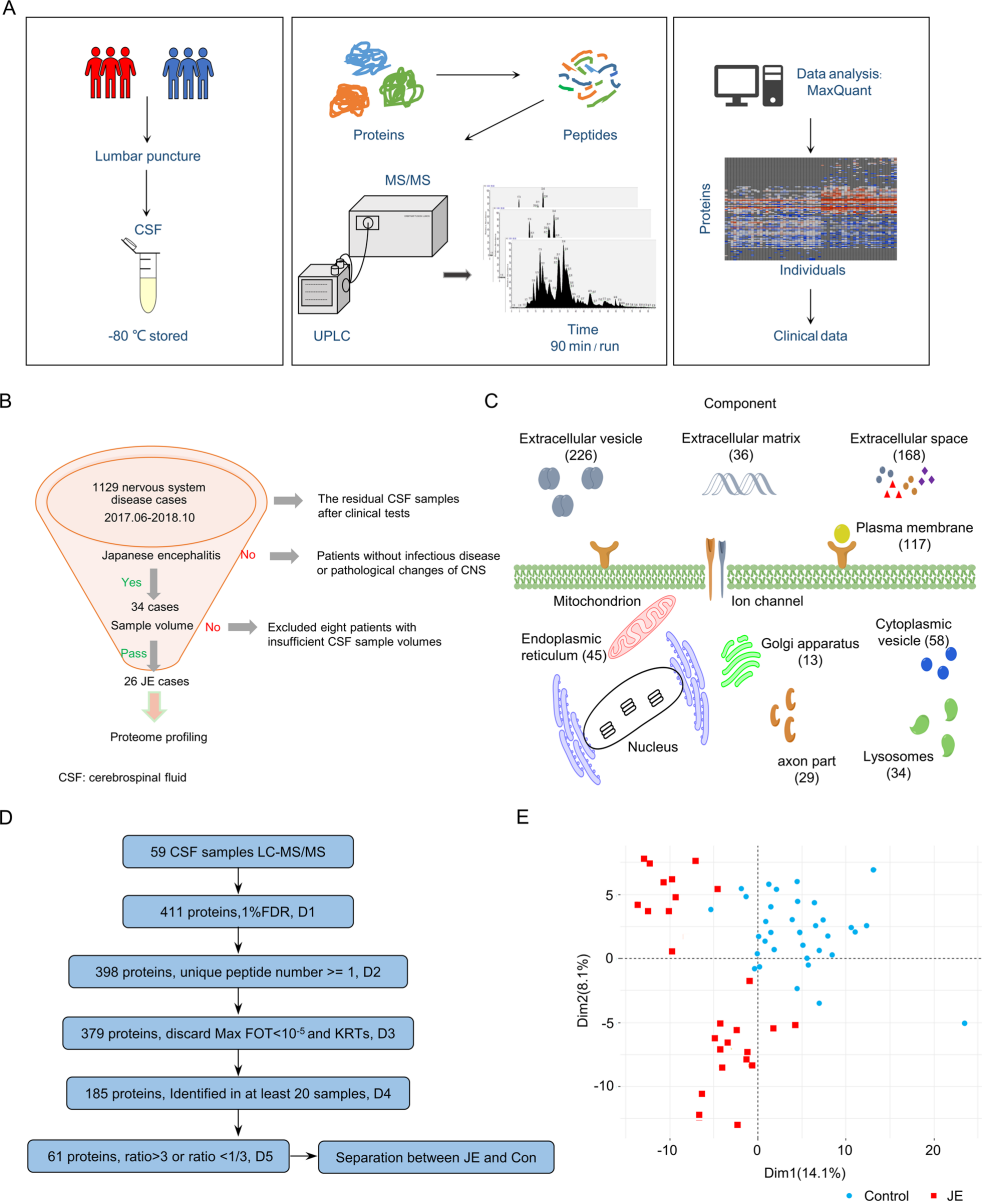
Overview of proteomics landscape of CSF in patients with JE. **A** Workflow of the quantitative proteomic analysis for CSF samples. **B** Enrolment of patients with JE and collection of clinical data. **C** Summary of proteomics data analysis procedure for CSF of patients with JE. **D** Proteomic data sets filtered at different levels for various statistical analyses. D1: 411 proteins were identified in 59 samples (26 patients and 33 controls) at 1% protein level FDR; D2: high confidence unique proteins identified with ≥ 1 unique peptides with peptide FDR < 1% that were used for identifying differentially expressed proteins (DEPs) between JE and control; D3: 379 identified proteins with high abundance range (FOT > 10 − 5 in at least one case) except potential KRT contaminants; D4: 185 proteins identified in more than one-third of the samples; D5: identified 59 differentially expressed proteins with FOT ratio JE/control > 3 or JE/control < 1/3; D6, Separation CSF sample between JE and Con. E. PCA score plot based on all detected proteins. Patients with JE (red) were apart from the control group (blue)

We collected CSF from 26 patients with JE and 33 control patients (Fig. 1A, B). Proteomics approach was used to evaluate the prognosis and possible mechanism of pathogenesis of cognitive impairments in patients with JE. The majority of identified proteins were extracellular proteins distributed in extracellular vesicles, extracellular space, or the extracellular matrix (Fig. 1C). Plasma membranes contained the second largest proportion of identified proteins, which suggested that the CSF data set may reflect the natural distribution of CSF proteins.

High-confidence unique proteins identified with unique peptides ≥ 1 and peptide FDR < 1% were used for identifying differentially expressed proteins between patients with JE and the control patients. We quantified 379 proteins with a high abundance range (filter output truncation (FOT) > 10^−5^ in at least one case) except potential Keratin contaminants (Fig. 1D). The range of variation of FOT values of identified proteins in different samples was similar (Additional file 1: Fig. S1E). In the data set, 327 proteins had at least 2 unique peptides (Additional file 2: Fig. S2A). Among them, 185 proteins were identified in more than one-third of the samples (Fig. 1D). Finally, we identified 61 differentially expressed proteins between patients with JE and control patients with FOT ratio ≥ 3 or ≤ 1/3, unique peptide ≥ 2, and *p* ≤ 0.05 (Fig. 1D, Additional file 8: Table S2, Additional file 9: Table S3). Proteome data from patients in the JE and control groups was divided into two clusters by principal component analysis (PCA) (Fig. 1E). These results indicated that patients with JE could be distinguished from control patients through a proteomic study.

### General features of the JE proteome

**Fig. 2:**
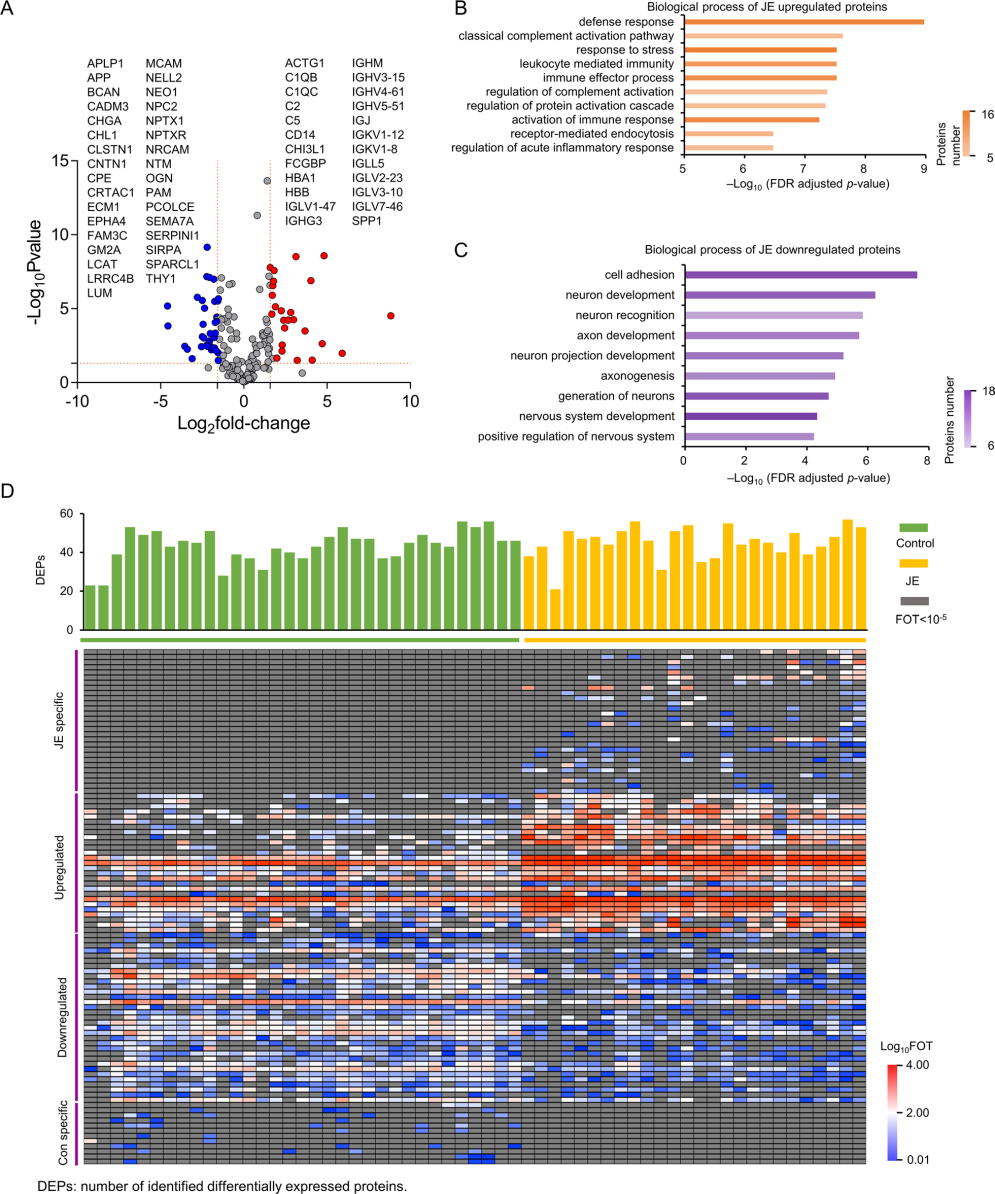
Differentially expressed proteins in CSF of patients with JE. **A** Association between protein expression (JE/Con ratio) and confidence level. A list of 25 overexpressed proteins with log-rank *P* value < 0.05 and log_2_ (JE/Con ratio) > 1.58 and 36 downregulated proteins with log-rank *P* value < 0.05 and log_2_ (JE/Con ratio) < − 1.58 in JE are included in the box on the left and right. **B** Biological process of JE upregulated proteins. **C** Biological process of JE downregulated proteins. **D** Heat map depicts the relative abundance of signature proteins (log_10_-transformed FOT). Change types related to these signature proteins are denoted on the left (detailed in Additional file 2: Table S2, Additional file 9: Table S3)

Quantitative proteomic analysis was performed using a label-free technique and on the same mass spectrometer with consistent quality control (Additional file 1: Fig. S1A), and identified 398 proteins from 59 CSF samples (Fig. 1D), as well as 25 JE upregulated or specific expressed proteins and 36 JE downregulated or unexpressed proteins (Fig. 2A, Additional file 2: Fig. S2B, Additional file 8: Table S2, Additional file 9: Table S3).

### Immune response proteins were upregulated in the CSF of JE

Significant differences were found between JE and control samples. Proteins with different expression were divided into two groups in heatmap analysis, including JE upregulated and JE specific proteins, JE downregulated and control specific proteins (Fig. 2A, D). Numerous antibody proteins were found upregulated in JE samples, included immunoglobulin J chain (IGJ) and Ig kappa chain V–I region (IGKV1–12) (Fig. 2A, Additional file 2: Fig. S2C, E, Additional file 3: Fig. S3A, B, Additional file 8: Table S2). Complement proteins were also found to upregulated in this group, included complement C1q subcomponent subunit B (C1QB) and complement C1q subcomponent subunit C (C1QC) (Fig. 2A). The upregulated proteins were primarily concentrated in pathways related to humoral immune response mediated by circulating immunoglobulins, lymphocyte-mediated immunity, and the classical complement activation pathway (Fig. 2B).

### Alzheimer’s disease (AD) related proteins changed in the CSF of JE

SPARCL1, Fibrinogen alpha chain precursor, Serum amyloid A-1 protein (SAA1), Amyloid β A4 precursor protein (APP), Amyloid-like protein 1 (APLP1), and Apolipoprotein E (APOE) precursor levels were different in patients with JE compared with control group (Fig. 2A, Additional file 2: Fig. S2D, F, Additional file 3: Fig. S3C, D, Additional file 9: Table S3). The levels of APLP1 and APP in the CSF of JE patients were lower than control group (*p* < 0.001, Table S3).

### Proteins regulating nervous development downregulated in the CSF of JE

Downregulated proteins in the CSF of patients with JE were primarily involved in regulation of nervous system development, including generation of neurons, neuron development, neuron recognition, axonogenesis, and axon development (Fig. 2C). In the CSF of patients with JE, the downregulated of neuron and axon development proteins indicated that the neuron and axon systems were damaged during JE infection (Fig. 2C, Additional file 9: Table S3).

### Patients with JE divided into two subtypes

**Fig. 3:**
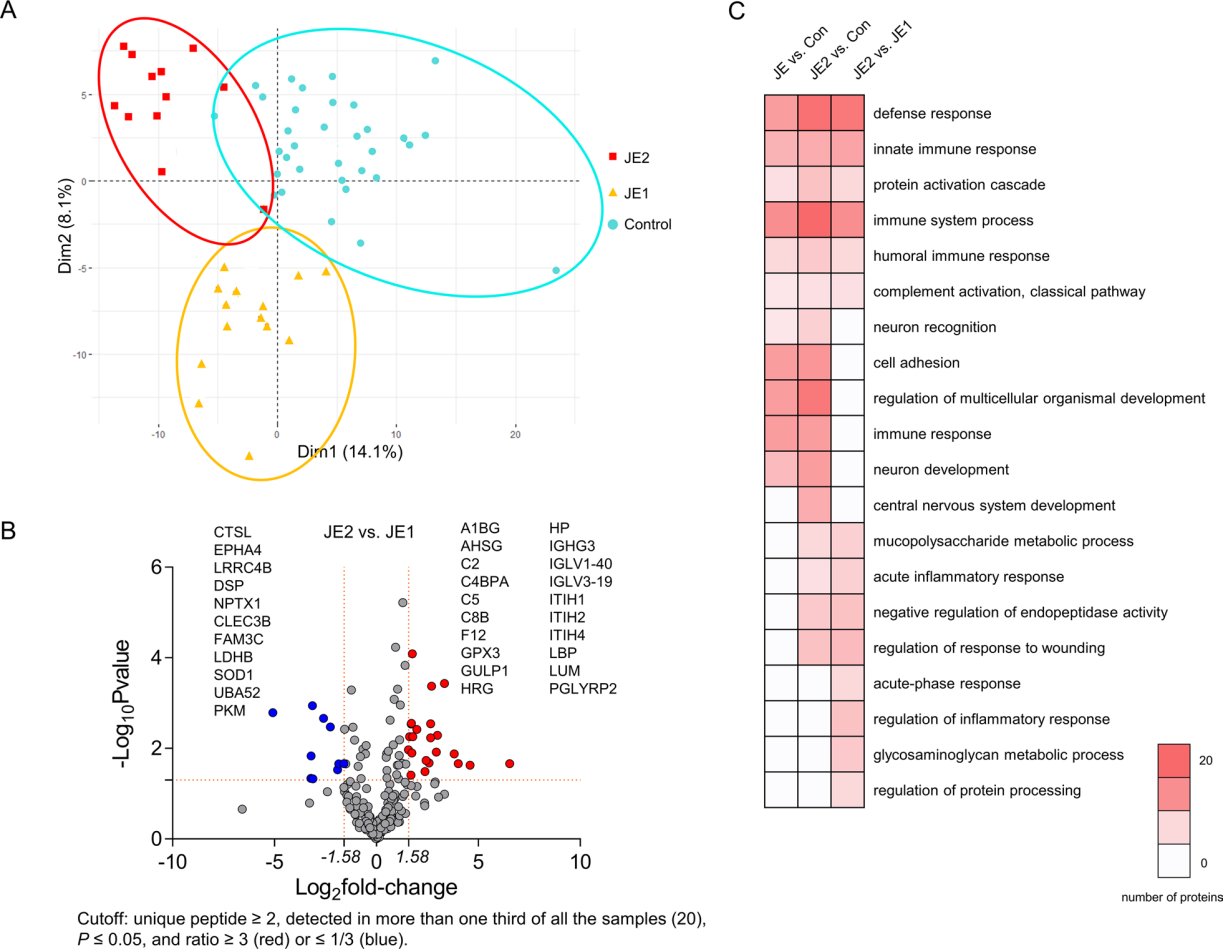
Changed proteins in CSF of patients with JE subgroups. **A** PCA score plot in all CSF samples based on detected proteins. Apart from the control group, all plots in JEI and JEII were clustered together, respectively, and each group (control, JEI, and JEII) could be easily differentiated. **B** Association between protein expression (JE/Con ratio) and confidence level. A list of 25 overexpressed proteins with log-rank *P* value < 0.05 and log_2_ (JE/Con ratio) > 1.58 and 36 downregulated proteins with log-rank *P* value < 0.05 and log_2_ (JE/Con ratio) < − 1.58 in JE are included in the box on the left and right. C. Differentially expressed proteins between JE2 and JE1 were mainly clustered on protein functions such as myeloid leukocyte-mediated immunity, antibody protein expression, and cell morphogenesis involved in neuron differentiation

Through the HCL analysis of the CSF proteomic data, the controls and patients with JE were clustered into three groups—control, JE1, and JE2 (Additional file 4: Fig. S4). The subtype grouping results were further verified by PCA, which also showed different distributions of the three groups consistent with the HCL results (Fig. 3A). The differentially expressed proteins between subtypes JE1 and JE2 were divided into two groups (Fig. 3B). There were 85 differentially expressed proteins between patients with subtype JE2 and JE1, among which 45 were upregulated and 40 were downregulated in the JE2 group (Additional file 10: Table S4, Additional file 11: Table S5). They were mainly clustered on protein functions such as myeloid leukocyte-mediated immunity, antibody protein expression, and cell morphogenesis involved in neuron differentiation (Fig. 3C).

### Immune response and peptidase activity related proteins were upregulated in JE2

**Fig. 4:**
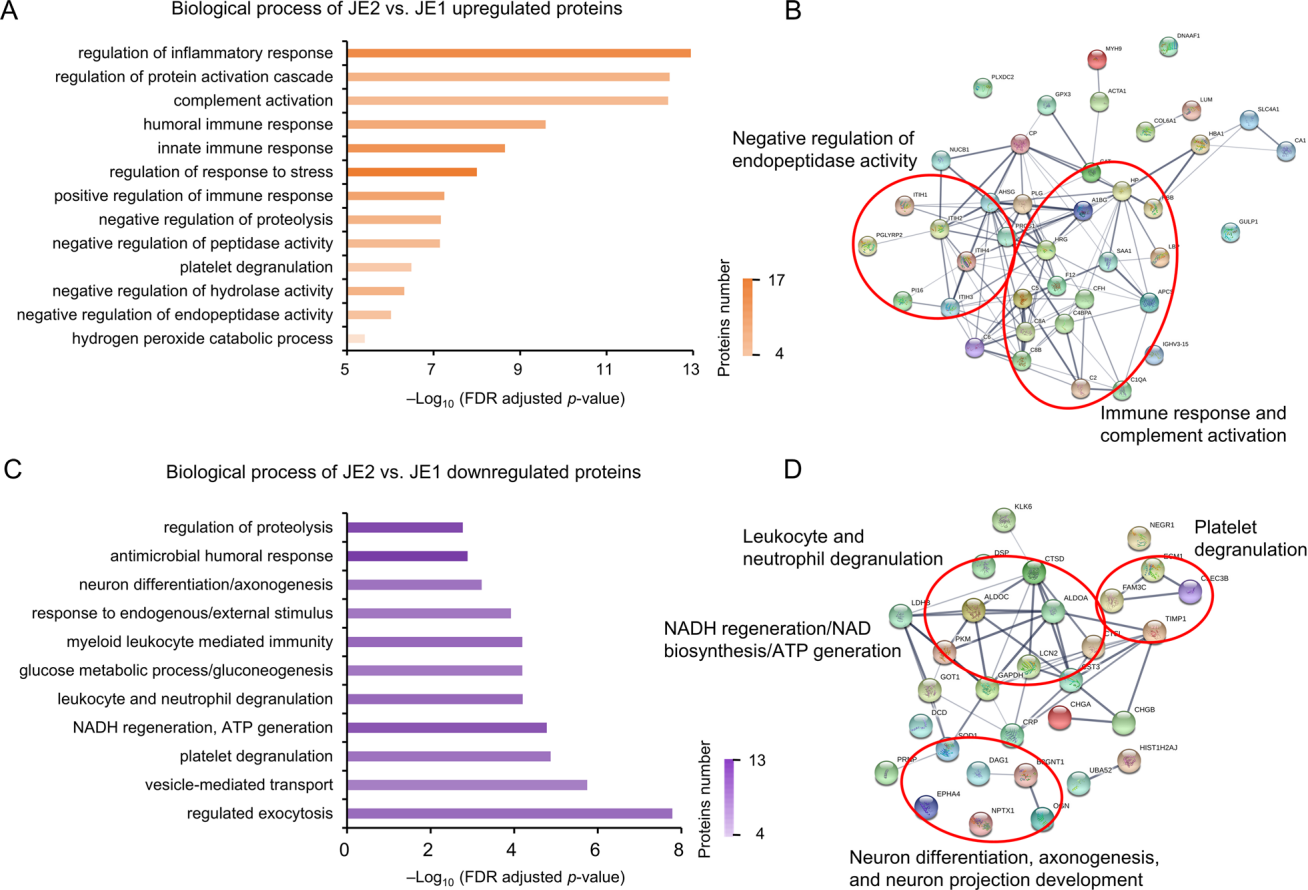
Changed proteins in CSF of patients in JE subgroups. **A** Biological process of JE2 vs. JE1 upregulated proteins based on GO analysis. **B** Protein interaction analysis of upregulated proteins evaluated using the STRING database. Negative regulation of endopeptidase activity, Immune response and complement activation proteins were the major upregulated proteins in JE2. **C** Biological process of JE2 vs. JE1 downregulated proteins based on GO analysis. **D** Neuron differentiation, axonogenesis, and neuron projection development, leukocyte and neutrophil degranulation, ATP generation, and platelet degranulation were the major upregulated proteins in JE2. **E** Heatmap of the differentially expressed proteins between subtypes JE1 and JE2

Proteins upregulated in the JE2 group were primarily involved in pathways associated with immune response and peptidase activity (Fig. 4A, B). The positively regulated or activated pathways included inflammation, immune response, complement activation, protein cascade activation, and response to stress. The negatively regulated pathways included proteolysis, peptidase activity, hydrolase activity, and platelet degranulation (Additional file 10: Table S4).

### Amyloid related proteins have changed in subgroup of JE

Amyloid-related protein expression changes were observed in JE subgroups. Among the amyloid-related proteins, SAA1 was only detected in 8 patients in the JE2 group (8 in 12, 66.6%), while none were detected in any control and JE1 group patients (Additional file 5: Fig. S5A). Serum amyloid P-component protein (APCS) was also detected in 50% of patients with JE2 (6 in 12), but not in any patient with JE1 (Additional file 5: Fig. S5B). APLP1 and APP levels were significantly lower in both JE1 and JE2 compared to control (*p* < 0.001, Additional file 5: Fig. S5C). The APP level in patients with JE2 has upregulated trend than in patients with JE1 (*p* = 0.073, Additional file 5: Fig. S5D).

### Proteins downregulated in JE2

Proteins downregulated in the JE2 group compared to the JE1 group influenced pathways including myeloid leukocyte-mediated immunity, NADH regeneration, NAD biosynthesis, ATP generation, gluconeogenesis, leukocyte, neutrophil, and platelet degranulation, cell morphogenesis involved in neuron differentiation, axonogenesis, and neuron projection development (Fig. 4C, D). The downregulated proteins in JE2 were associated with myeloid leukocyte-mediated immunity, leukocyte and neutrophil degranulation (Additional file 11: Table S5).

### Clinical symptoms and prognosis were different between the JE1 and JE2 groups

**Fig. 5:**
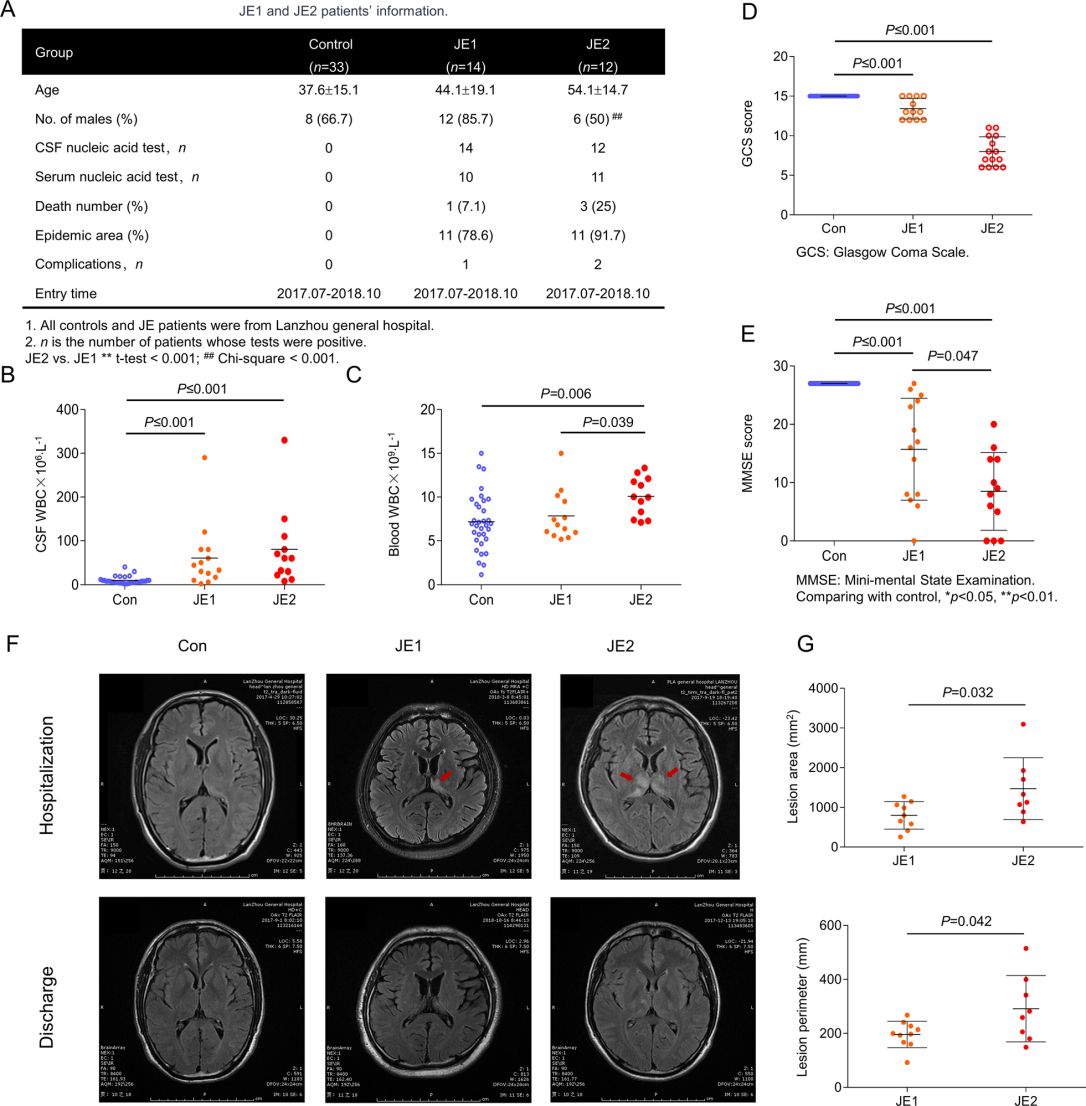
Clinical symptoms and prognosis in patients in different JE subgroups. **A** Patients’ basic information for the two JE subtypes. **B** CSF WBC count was significantly higher in JE subgroups compared to the control group. **C** WBC count in the blood was significantly higher in patients with JE2 compared to that in patients with JE1. **D** GCS scores of patients in three groups. **E** MMSE scores of patients in three groups. **F** MRI examinations in the fluid-attenuated inversion-recovery mode showed a clear distribution of lesions around the caudate nucleus in patients with JE. **G** Lesion area and perimeter of infection were larger in different JE subgroups

Patient information for the two JE subtypes is shown in Fig. 5A. DNA tests for JEV in the CSF were all positive in the two groups. The CSF WBC count was significantly higher in both JE1 and JE2 groups compared to the control group. However, there was no significant difference in CSF WBC counts between the patients in the JE subgroups (Fig. 5B). The WBC count in the blood was significantly higher in patients with JE2 compared to that in patients with JE1 (Fig. 5C). The GCS scores were significantly lower in patients with JE1 and JE2 than in the control patients. However, there was no difference in the GCS scores of the JE1 and JE2 groups (Fig. 5D). The MMSE scores were also significantly lower in patients in the JE1 and JE2 groups compared to those of the control group. MMSE scores in patients with JE2 (8.5 ± 6.7) were lower than in patients with JE1 (15.7 ± 8.7) before discharge (Fig. 5E). MRI examinations in the fluid-attenuated inversion-recovery mode showed a clear distribution of lesions around the caudate nucleus in patients with JE1 and JE2 (Fig. 5F). The lesion area and perimeter of infection were larger in the JE2 group than in the JE1 group (Fig. 5G). The blood WBC count in patients with JE2 (10.08 ± 2.21, 10^9^·L^−1^) was higher than in patients with JE1 (7.84 ± 2.20, 10^9^·L^−1^, *p* = 0.039), and the RBC count in patients with JE2 (4.39 ± 0.50, 10^12^·L^−1^) was also higher than in patients with JE1 (3.91 ± 0.56, 10^12^·L^−1^, *p* = 0.034). There was no significant difference in other tests between the two groups.

### The percentages of N%, L%, and M% decreased significantly in JE2

**Fig. 6:**
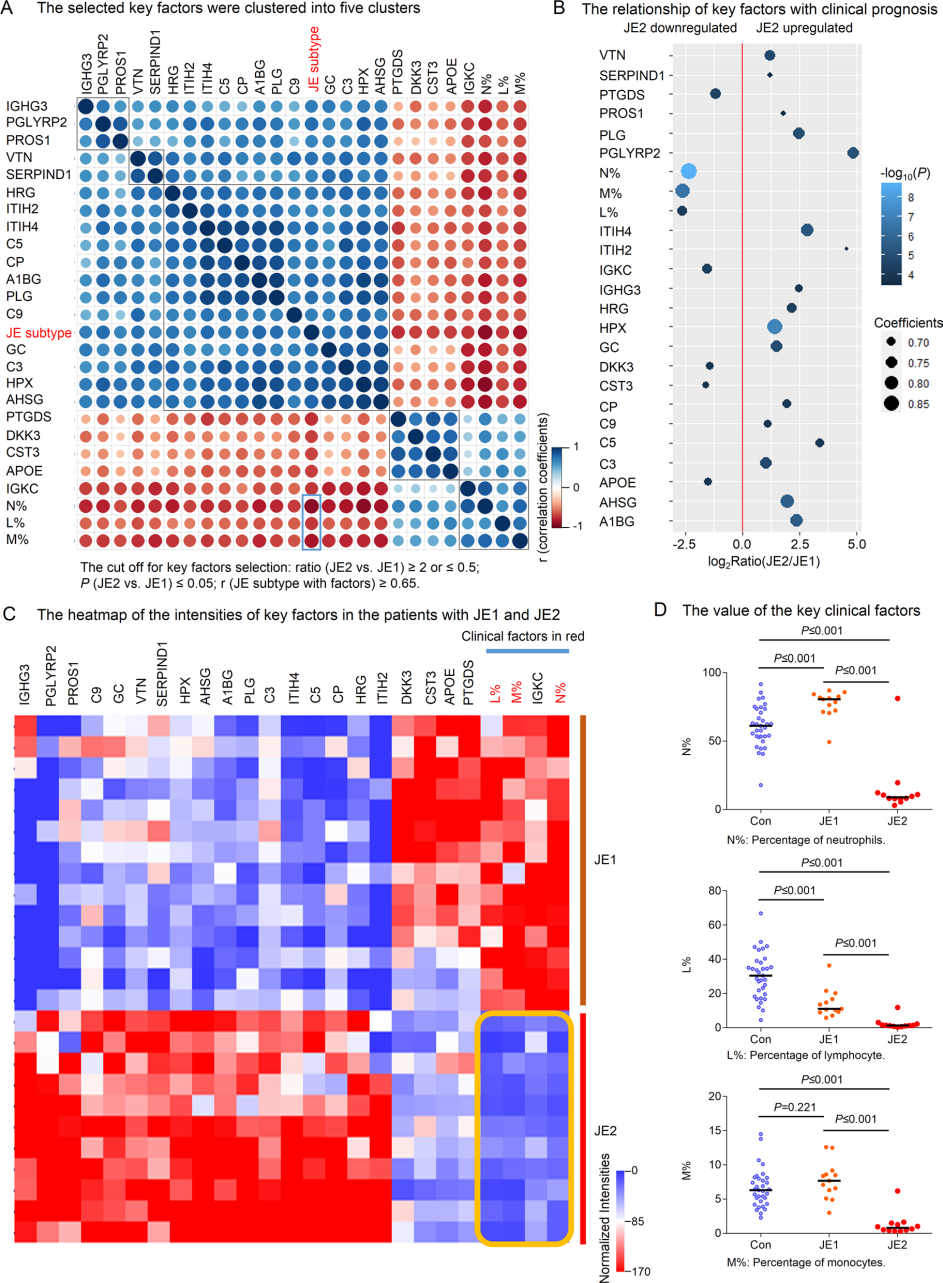
Correlation of different clinical indicators and proteins in patients in different JE subgroups. **A** Selected 25 key factors were clustered into five clusters. **B** Relationship of key factors with clinical prognosis. **C** Heatmap of the intensities of 25 key factors in the patients with JE1 and JE2. **D** Value of the key clinical factors

The different proteomics proteins and clinical indicators between JE1 and JE2 were analysed. The HCL cluster and correlative analysis were performed. The results showed that the levels of N%, L%, and M% were negatively correlated with humoral immune proteins, such as IGHG3, Complement C3 (C3), Complement C5 (C5), Complement C9 (C9), and Ceruloplasmin (CP) (Fig. 6A). The cutoff of changed proteins included in correlative analysis: ratio (JE2 vs. JE1) ≥ 2 or ≤ 0.5, and *P* (JE2 vs. JE1) ≤ 0.05; r (JE subtype with each factors) ≥ 0.65. The cutoff of changed clinical factors included in correlative analysis: *P* (JE2 vs. JE1) < 0.05 (Fig. 6A). Complement components C3, C5, C9, and CP were significantly upregulated in CSF of JE2 compared with JE1 (Fig. 6B). The levels of N%, L%, and M% were significantly downregulated in CSF of JE2 compared with JE1. The proteins decreased in JE2 compared with JE1 were APOE, Cystatin C (CST3), DKK3, and PTGDS (Fig. 6B). JE1 and JE2 showed different heatmap profiling (Fig. 6C). The value of Percentage of neutrophils, lymphocyte, and monocytes showed significant difference in JE2 compared with JE1 (Fig. 6D).

## Discussion

At present, the difficulty of severe JE therapy research is that the prognosis markers and treatment targets are unclear. The clinical treatment is mainly focused on life support, lacking of targeted treatment strategy. It is challenging but of pressing need to integrate of large sample size, high quality clinical data, and large-scale omics data. In present study, the combined clinical and proteomics data of patients with JE was analysed for the first time.

The comparison of CSF proteomics profiling between patients with severe JE and control group is just one aspect of this study. Another key point is to study the clinical and molecular differences between different prognoses of severe JE patients. The study explored the correlation within prognosis and various clinical indicators.

The patients enrolled in this study were all severe patients. The upregulated pathways in patients with JE were mainly concentrated in the humoral immune response, lymphocyte-mediated immunity, and the classical complement activation pathway. These results were consistent with the CSF white blood cell (WBC) results, indicating that the cellular immune response system and complement activation system were activated in the CSF of patients with JE. Numerous antibodies and complement proteins were also found upregulated in JE, which was congruent with the general understanding of JE infection.

It was still unclear how amyloid related proteins took part in the cognitive impairment during JE progress. Previous paper reported that APP distributed differently with β-Amyloid in human tissue [27]. Our results showed the different expression trends for β-Amyloid and Amyloid beta A4 precursor protein in the CSF of patients’ with JE. APP was downregulated in the CSF of JE patients, while β-Amyloid was upregulated (Additional file 6: Figure S6A). Among the amyloid related proteins, most patients in JE2 have detected serum amyloid A-1 (SAA1) protein in CSF, and SAA1 was only detected in the JE2 patients. Recent in vivo study reported overexpression of SAA1 intensified the neuronal inflammation in amyloid abundant condition and caused greater memory decline in amyloid beta A4 protein (APP)/SAA1 transgenic mice [28]. The difference of SAA1 level in CSF might be relative with different dementia symptoms for JE patients, which need to be paid more attention in future research. The APP level in patients with JE2 has upregulated trend than in patients with JE1.

The subtype of JE (JE1 and JE2) showed different proteomic profiling. The proteomic trends were different in JE1, JE2, and control groups. Cellular immunity and humoral immunity play key roles in different prognosis of patients with JE. Besides the classical pathway complement components, the late complement components were also upregulated in patients in the JE2 compared to those in the JE1. These results showed that the classical complement pathway, and not the alternative pathway, was activated in JEV infection. The upregulation of the late complement components indicated potential poor prognosis of patients with JE.

There were more complement activation proteins upregulated in JE2 compared with JE1. The complement system activation is involved in the pathogenesis of a variety of neurodegenerative diseases, such as Alzheimer’s disease, Parkinson’s disease and multiple sclerosis [29]. West Nile virus (WNV) infection of adult hippocampal neurons induces complement-mediated elimination of presynaptic terminals in a murine WNV neuroinvasive disease model. C1QA was upregulated and localized to microglia, infected neurons and presynaptic terminals during WNV neuroinvasive disease. Complement activation may act as an intermediate between β/A4 deposits and the neurotoxicity observed in AD [30]. In our results, we found besides the classical pathway complement components C1QA, C2, C4BPA, the late complement components C5, C6, C8A, C8B, and the complement regulator CFH were also upregulated in JE2 compared with JE1 (Additional file 6: Fig. S6B, Additional file 10: Table S4). However, C3B was not observed to be upregulated in CSF of JEV infection. These results indicated that the classical complement activation pathway but not the alternative pathway was activated in JEV infection. The membrane attack complex (MAC) consisted of the complement components C5b, C6, C8A, and C8B. Their upregulation indicated the activation of MAC in CSF of JE patients resulting in lysis of the target cell [31]. The upregulation of the late complement components might indicate potential poor prognosis of JE. Our results suggest that the late complement components levels might be used as a prognostic marker for cognitive impairment in the sequelae of JEV infection.

APOE has been implicated in Alzheimer’s disease, atherosclerosis, and other unresolvable inflammatory conditions but a common mechanism of action remains elusive [32]. APOE was reported to attenuate unresolvable inflammation as a checkpoint by complex formation with activated C1q in the complement activation pathway and subsequently reducing C5 and the inflammatory burden in atherosclerosis and Alzheimer disease [32]. In our study APOE was associated with JE cognitive sequelae (Fig. 6B). It was downregulated, while C5a was upregulated in patients with JE2 subtype which had poor cognitive impairment prognostic (Additional file 6: Fig. S6B, C).

Acute-phase response proteins as Inter-alpha-trypsin inhibitor Heavy Chains (ITIHs) upregulation partially reflected the activation of humoral immunity [33]. We found four isoforms ITIH1, ITIH2, ITIH3, and ITIH4 showed differently expressed pattern between JE1 and JE2 (Additional file 10: Table S4). ITIHs have been reported to attenuate complement activation and complement-induced lung injury [34]. The increased ITIH isoforms were correspondent with upregulated complement components. The upregulation of ITIH isoforms might be taken as a marker of poor clinical outcome in JE patients.

## Conclusions

In conclusion, we have identified two new subtypes of JE associated with significantly different prognoses. The differences in proteomic landscape between the JE subgroups have different prognosis of cognitive impairment. The decreased levels of N%, L%, and M% and levels of increased complement components can be considered as clinical markers for poor prognosis of JE. Increased level of APOE, CST3, DKK3, and PTGDS can be considered as molecular markers for good prognosis of JE. The CSF proteomic classification of JE provides new evidence for understanding the pathogenesis of JE and the relationship of JEV infection with dementia symptoms.

## Supplementary Information

**Additional file 1: Figure S1.** MS platform quality control and CSF protein identifications. A. Pairwise scatter plots and Spearman’s correlation coefficients for replicate proteome profiling of eight CSF samples. Notably, repeat experiments with the same samples have good reproducibility, with a high level of correlation (average, 0.977; range, 0.966–0.990). B. Cumulative number of peptides identified as a function of CSF sample numbers. Cumulative number of proteins identified as a function of CSF sample numbers. C. Cumulative number of peptides identified as a function of CSF loading amount. Cumulative number of proteins identified as a function of CSF loading amount. D. Results of quantified proteins in JE and control CSF samples. Total and average number of proteins quantified in each case. E. Distribution of log10 transformed FOT of the identified proteins in 59 CSF samples.

**Additional file 2: Figure S2.** Proteomic features of the JE. A. Summary of MS identification and quantitation. B. Differentially expressed proteins among three groups. C. Cellular component of JE upregulated proteins. D. Cellular component of JE downregulated proteins. E. Significantly increased proteins in JE CSF samples. The *y*-axis represents log10FOT. F. Significantly decreased proteins in JE CSF samples. The *y*-axis represents log10FOT. ***: p < 0.001.

**Additional file 3: Figure S3.** Western blot of classical JE changed proteins. Each three to four control or JE samples were randomized pooled into one mixed sample. A. Western blot of Ig λ chain. B. Western blot of Ig E. C. Western blot of SPARCL1. D. Western blot of β57 Amyloid. The relative gray intensities for the western blot results of Ig λ chain (E), Ig E chain (F), SPARC-like 1 protein (SPARCL1) (G), and β-Amyloid (H) were calculated.

**Additional file 4: Figure S4.** Through Hierarchical clustering (HCL) analysis, the 59 samples were mainly clustered into 3 groups, as control, JE1, and JE2.

**Additional file 5: Figure S5.** Amyloid related proteins has changed in patients with JE. The proteomics results for the expression of Serum amyloid A-1 protein (SAA1) (A), Serum amyloid P-component protein (APCS) (B), Amyloid-like protein 1 (APLP1) (C), and Amyloid β A4 precursor protein (APP) (D) in patients with JE are shown. FOT: normalization to fraction of total.

**Additional file 6: Figure S6.** Western blot validation results. A. The different trends of expression for Amyloid A4 precursor and β-Amyloid in the CSF of patients’ with JE. B. The expression for C5a in the CSF of patients’ with subtype JE C. The expression for APOE in the CSF of patients’ with subtype JE. CSF: cerebrospinal fluid; C5a: Complement C5a; APOE: Apolipoprotein E. Each loading samples were mixed by four CSF samples of patients in control and patients with JE1 or JE2.

**Additional file 7: Table S1.** The demographics and clinical features of the patients.

**Additional file 8: Table S2.** Upregulated proteins related with JE.

**Additional file 9: Table S3.** Downregulated proteins related with JE.

**Additional file 10: Table S4.** Upregulated proteins in JE2 comparing with JE1.

**Additional file 11: Table S5.** Downregulated proteins in JE2 comparing with JE1

## Data Availability

The mass spectrometry proteomics data have been deposited to the ProteomeXchange Consortium via the iProX partner repository [35] with the data set identifier PXD021697. All other data are available in the main text or the additional materials.

## Abbreviations

A1BG: α-1B-glycoprotein
ACN: Acetonitrile
AD: Alzheimer’s disease
AHSG: α-2-HS-glycoprotein
ALDOA: Fructose-bisphosphate aldolase A
ALDOC: Fructose-bisphosphate aldolase C
APCS: Serum amyloid P-component
APLP1: Amyloid-like protein 1
APP: Amyloid β A4 precursor protein
BBB: Blood–brain barrier
C1QA: Complement activation proteins complement C1q subcomponent subunit A
C1QB: Complement C1q subcomponent subunit B
C1QC: Complement C1q subcomponent subunit C
C2: Complement C2
C4BPA: C4b-binding protein α chain
C5: Complement C5
C6: Complement component C6
C8A: Complement component C8 alpha chain
C8B: Complement component C8 β chain
CNS: Central nervous system
CP: Ceruloplasmin
CSF: Cerebrospinal fluid
F12: Coagulation factor XII
FDR: False discovery rate
HCL: Hierarchical Clustering
HP: Haptoglobin
HRG: Histidine-rich glycoprotein
iBAQ: Intensity-based absolute quantification
IGHA1: Ig alpha-1 chain C region
IGHG3: Ig gamma-3 chain C region
IGHM: Ig mu chain C region
IGHV4–61: Ig heavy chain V–II region ARH-77
IGHV5–51: Immunoglobulin heavy variable 5–51
IGJ: Immunoglobulin J chain
IGKV1–12: Ig kappa chain V–I region Daudi
IGLL5: Immunoglobulin lambda-like polypeptide 5
IGLV3–10: Ig lambda chain V–IV region Bau
IGLV7–46: Ig lambda chain V region 4A
ITIH1: Inter-α-trypsin inhibitor heavy chain-1
ITIH2: Inter-α-trypsin inhibitor heavy chain-2
ITIH4: Inter-α-trypsin inhibitor heavy chain-4
JE: Japanese encephalitis
JEV: Japanese encephalitis virus
LBP: Lipopolysaccharide-binding protein
LC–MS/MS: Liquid chromatography with tandem mass spectrometry
MS: Mass spectrometry
MAC: Membrane attack complex
MMSE: Mini-Mental State Exam
MRI: Magnetic resonance imaging
mRS: Modified Rankin Score
PCA: Principal component analysis
PGLYRP2: N-acetylmuramoyl-l-alanine amidase
PKM: Pyruvate kinase PKM
RBC: Red blood cell
SAA1: Serum amyloid A-1 protein
SOD1: Cu–Zn superoxide dismutase
SPARCL1: SPARC-like 1 protein
WBC: White blood cell
