# Supplementary figures and images for: Proteomic landscape subtype and clinical prognosis of patients with the cognitive impairment by Japanese encephalitis infection
Source: J Neuroinflammation. 2022 Apr 4;19:77. doi: 10.1186/s12974-022-02439-5 (PMC8981687; doi:10.1186/s12974-022-02439-5)

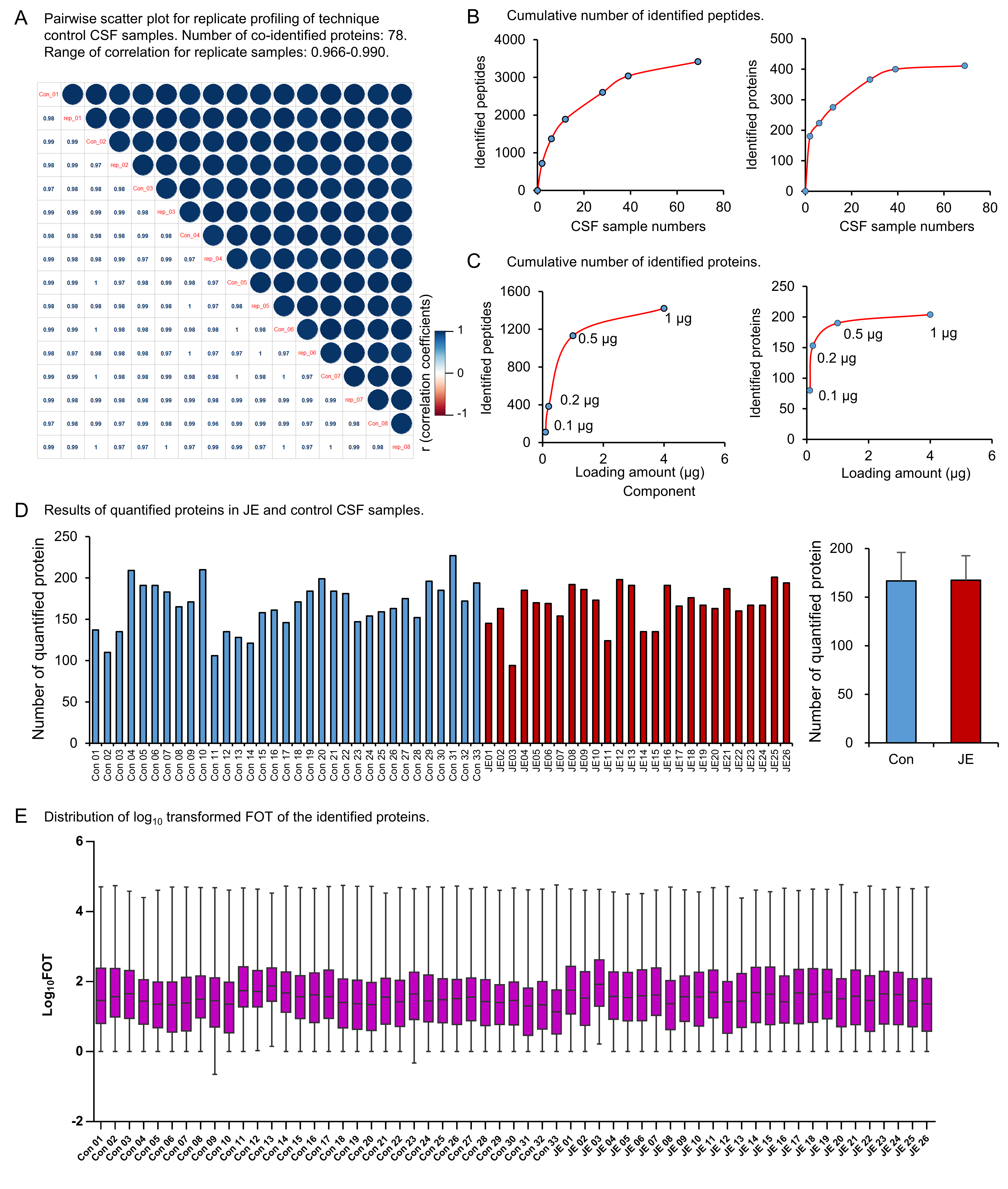

Supplement: Supplementary file 1 — Additional file 1: Figure S1. MS platform quality control and CSF protein identifications. A. Pairwise scatter plots and Spearman’s correlation coefficients for replicate proteome profiling of eight CSF samples. Notably, repeat experiments with the same samples have good reproducibility, with a high level of correlation (average, 0.977; range, 0.966–0.990). B. Cumulative number of peptides identified as a function of CSF sample numbers. Cumulative number of proteins identified as a function of CSF sample numbers. C. Cumulative number of peptides identified as a function of CSF loading amount. Cumulative number of proteins identified as a function of CSF loading amount. D. Results of quantified proteins in JE and control CSF samples. Total and average number of proteins quantified in each case. E. Distribution of log10 transformed FOT of the identified proteins in 59 CSF samples. [file 12974_2022_2439_MOESM1_ESM.tif]

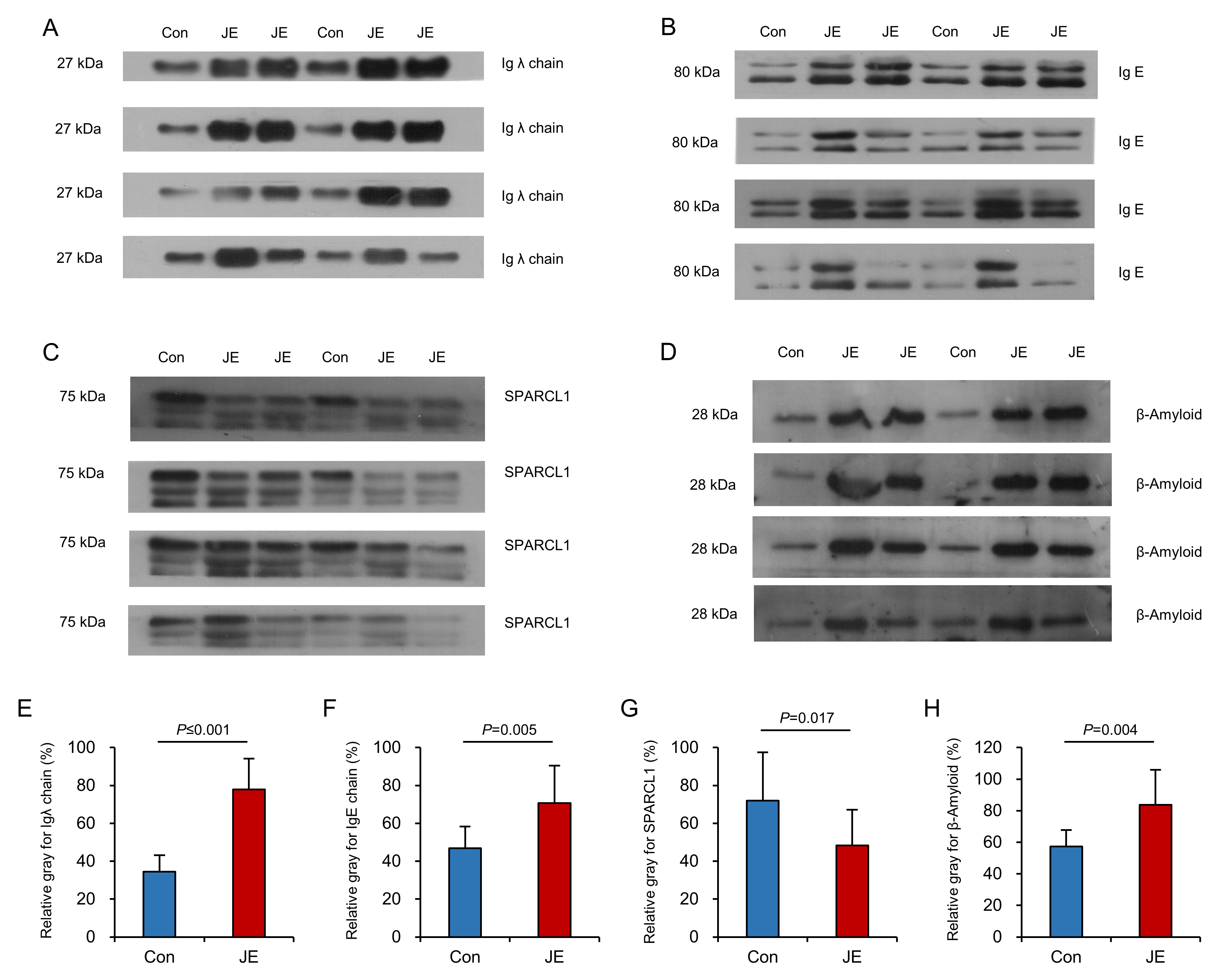

Supplement: Supplementary file 3 — Additional file 3: Figure S3. Western blot of classical JE changed proteins. Each three to four control or JE samples were randomized pooled into one mixed sample. A. Western blot of Ig λ chain. B. Western blot of Ig E. C. Western blot of SPARCL1. D. Western blot of β57 Amyloid. The relative gray intensities for the western blot results of Ig λ chain (E), Ig E chain (F), SPARC-like 1 protein (SPARCL1) (G), and β-Amyloid (H) were calculated. [file 12974_2022_2439_MOESM3_ESM.tif]

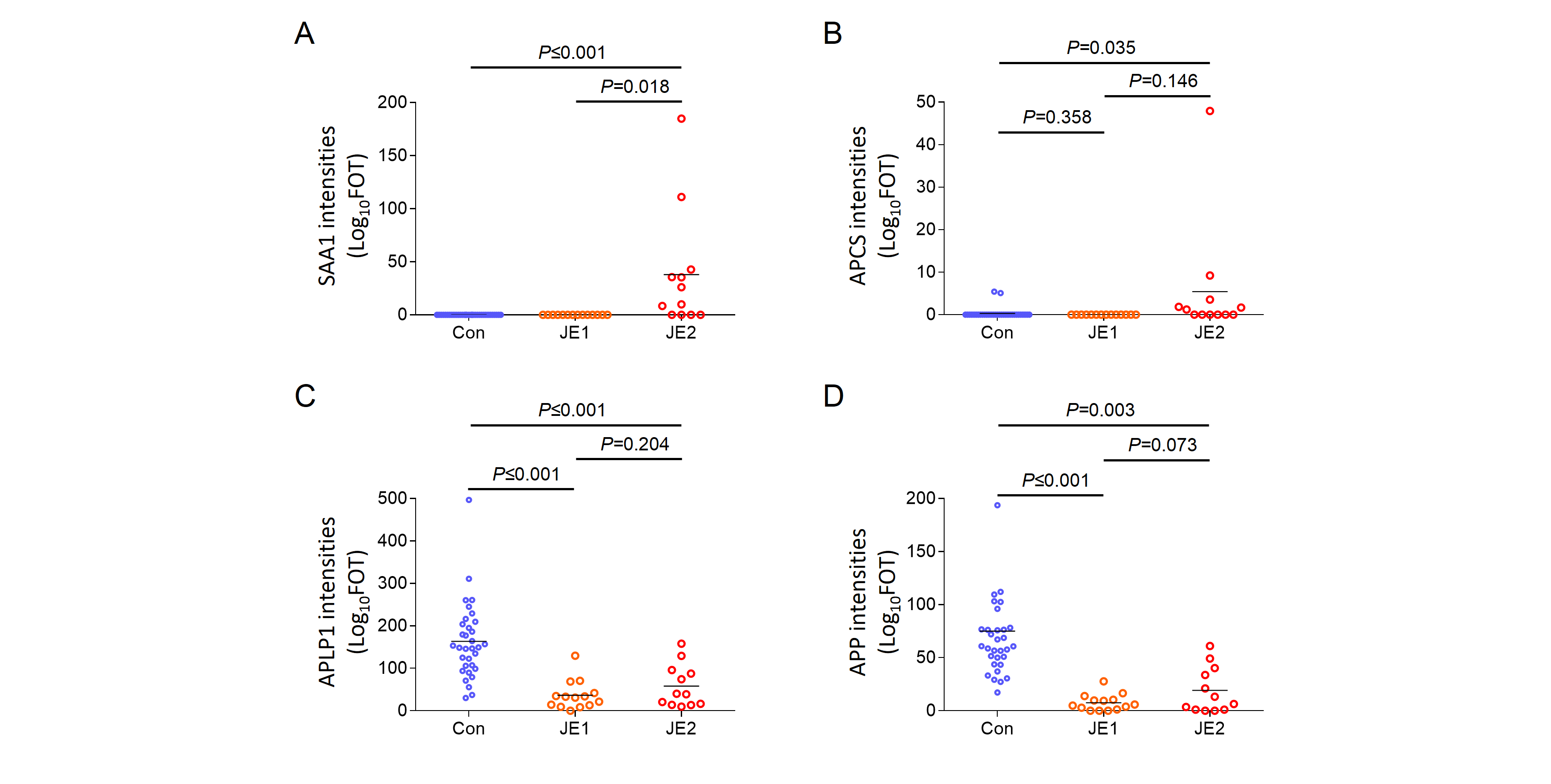

Supplement: Supplementary file 5 — Additional file 5: Figure S5. Amyloid related proteins has changed in patients with JE. The proteomics results for the expression of Serum amyloid A-1 protein (SAA1) (A), Serum amyloid P-component protein (APCS) (B), Amyloid-like protein 1 (APLP1) (C), and Amyloid β A4 precursor protein (APP) (D) in patients with JE are shown. FOT: normalization to fraction of total. [file 12974_2022_2439_MOESM5_ESM.tif]

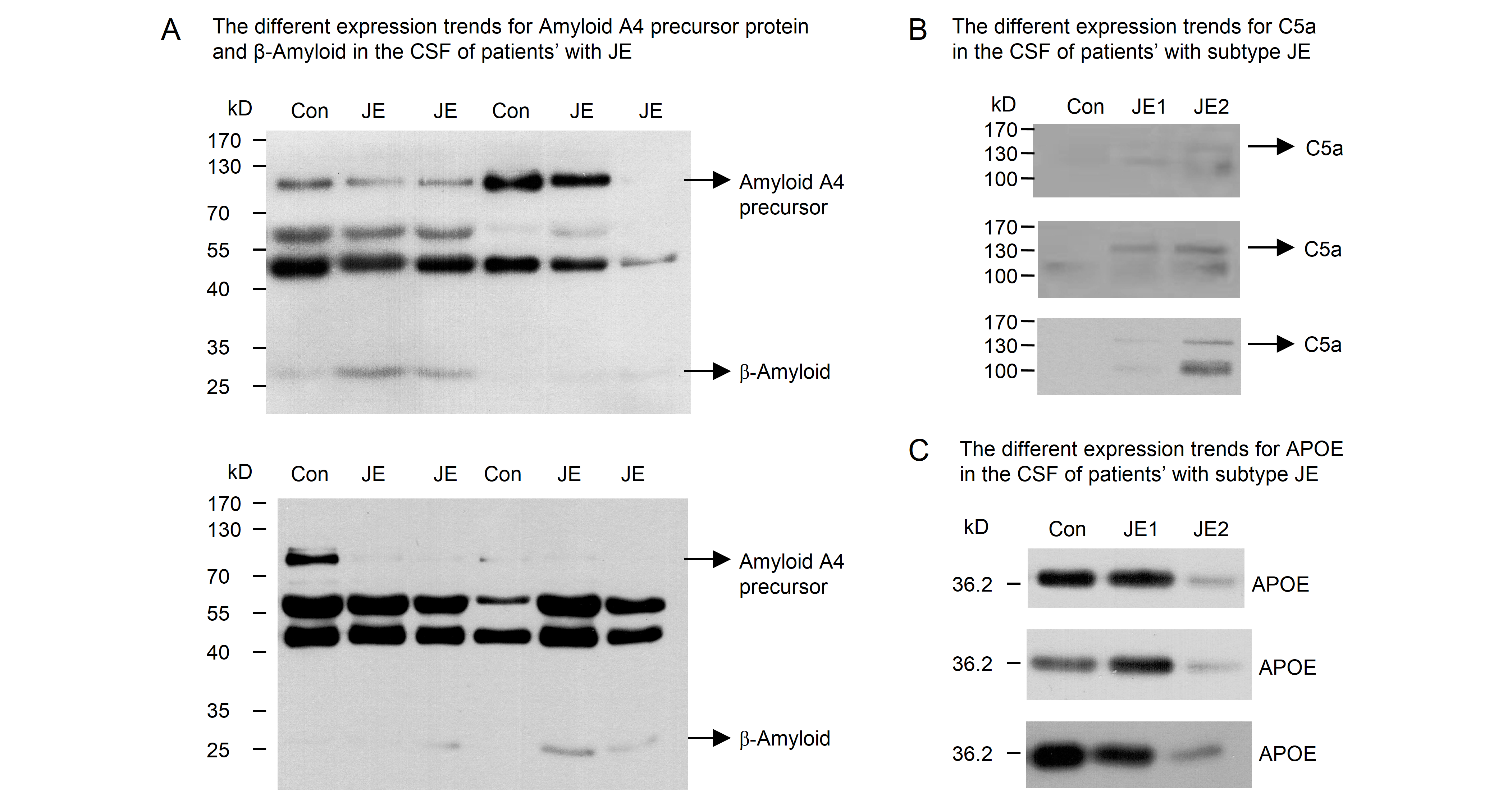

Supplement: Supplementary file 6 — Additional file 6: Figure S6. Western blot validation results. A. The different trends of expression for Amyloid A4 precursor and β-Amyloid in the CSF of patients’ with JE. B. The expression for C5a in the CSF of patients’ with subtype JE C. The expression for APOE in the CSF of patients’ with subtype JE. CSF: cerebrospinal fluid; C5a: Complement C5a; APOE: Apolipoprotein E. Each loading samples were mixed by four CSF samples of patients in control and patients with JE1 or JE2. [file 12974_2022_2439_MOESM6_ESM.tif]
