# Supplementary material for: Proteomic landscape subtype and clinical prognosis of patients with the cognitive impairment by Japanese encephalitis infection
Source: J Neuroinflammation. 2022 Apr 4;19:77. doi: 10.1186/s12974-022-02439-5 (PMC8981687; doi:10.1186/s12974-022-02439-5)
Supplement: Supplementary file 7 — Additional file 7: Table S1. The demographics and clinical features of the patients. [file 12974_2022_2439_MOESM7_ESM.pdf]

Table S1: The demographics and clinical features of the patients.

Characteristics (*n*=59)	%, median or range	control (*n*=33)	JE (*n*=26)	*p*-value
Basic profile				
Male	37 (62.7%)	19 (57.6%)	18 (69.2%)	0.358
Median age	42.5 (16-72)	37.5 (16-70)	48.5 (19-72)	0.012
Age subgroup				
>60	15 (25.4%)	3 (9.1%)	12 (46.2%)	
50-59	6 (18.2%)	4 (12.1%)	2 (7.7%)	
40-49	12 (20.3%)	8 (24.2%)	4 (15.4%)	
30-39	8 (13.6%)	5 (15.2%)	3 (11.5%)	
20-29	11 (18.6%)	9 (27.3%)	2 (7.7%)	
10-19	7 (11.9%)	4 (12.1%)	3 (11.5%)	
Living area				
Epidemic area	27 (45.8%)	5 (15.2%)	22 (84.6%)	
Other area	32 (54.2%)	28 (84.8%)	4 (15.4%)	
Central respiratory failure	12 (20.3%)	0 (0%)	12 (46.2%)	<0.001
Body temperature	38 (36.5-41)	37.3 (36.5-40)	38.9 (36.7-41)	<0.001
Unconsciousness	19 (32.2%)	0 (0%)	19 (73.1%)	<0.001
Seizure	3 (5.1%)	0 (0%)	3 (11.5%)	0.045
Mental symptoms	18 (30.5%)	0 (0%)	18 (69.2%)	<0.001
Limb paralysis	21 (35.6%)	8 (24.2%)	13 (50.0%)	0.040
Lung infection	19 (32.2%)	3 (9.1%)	16 (61.5%)	<0.001
Pathologic reflex	15 (51.7%)	3 (9.1%)	12 (46.2%)	0.0012
MRI abnormal	17 (28.8%)	2 (6.1%)	15 (57.7%)	<0.001
Death	4 (6.8%)	0 (0%)	4 (15.4%)	0.0196
Blood test				
WBC (10^9/L)	7.88±3.08	7.17±3.17	8.78±2.72	0.048
RBC (10^{12}/L)	4.45±0.82	4.68±0.91	4.15±0.56	0.013
Calcitonin original (ng/mL)	0.63±1.68	0.12±0.14	1.13±2.23	0.035
Blood glucose (mmol/L)	6.17±2.31	5.34±1.31	7.22±2.82	0.001
CSF test				
Intracranial pressure (mmH_2O)	164.8±61.0	158.5±59.6	172.84±61.80	0.38
WBC (10^6/L)	54.4±161.7	9.67±8.13	111.23±231.30	0.016
Glucose (mmol/L)	3.56±0.96	3.46±0.47	3.69±1.34	0.36
Chloride content(mmol/L)	124.9±6.1	127.2±2.8	121.9±7.7	<0.001
Protein (mg/L)	484.5±287.7	329.9±173.1	680.7±284.4	<0.001
GCS	13 (3-15)	15 (15)	10 (3-15)	<0.001
MMSE	22.1±8	27±0	15.7±8.7	<0.001
MRS				
0	8 (13.6%)	0 (0%)	8 (30.8%)	

1	11 (18.6%)	0 (0%)	11 (42.3%)
2	3 (5.1%)	0 (0%)	3 (11.5%)
3-5	0	0	0
6	4 (6.8%)	0 (0%)	4 (15.4%)
